# NOD1^CARD^ Might Be Using Multiple Interfaces for RIP2-Mediated CARD-CARD Interaction: Insights from Molecular Dynamics Simulation

**DOI:** 10.1371/journal.pone.0170232

**Published:** 2017-01-23

**Authors:** Jitendra Maharana, Sukanta Kumar Pradhan, Sachinandan De

**Affiliations:** 1 Department of Bioinformatics, Orissa University of Agriculture and Technology, Bhubaneswar, Odisha, India; 2 Animal Genomics Lab., Animal Biotechnology Centre, ICAR-National Dairy Research Institute, Karnal, Haryana, India; Koç University, TURKEY

## Abstract

The nucleotide-binding and oligomerization domain (NOD)-containing protein 1 (NOD1) plays the pivotal role in host-pathogen interface of innate immunity and triggers immune signalling pathways for the maturation and release of pro-inflammatory cytokines. Upon the recognition of iE-DAP, NOD1 self-oligomerizes in an ATP-dependent fashion and interacts with adaptor molecule receptor-interacting protein 2 (RIP2) for the propagation of innate immune signalling and initiation of pro-inflammatory immune responses. This interaction (mediated by NOD1 and RIP2) helps in transmitting the downstream signals for the activation of NF-κB signalling pathway, and has been arbitrated by respective caspase-recruitment domains (CARDs). The so-called CARD-CARD interaction still remained contradictory due to inconsistent results. Henceforth, to understand the mode and the nature of the interaction, structural bioinformatics approaches were employed. MD simulation of modelled 1:1 heterodimeric complexes revealed that the type-Ia interface of NOD1^CARD^ and the type-Ib interface of RIP2^CARD^ might be the suitable interfaces for the said interaction. Moreover, we perceived three dynamically stable heterotrimeric complexes with an NOD1:RIP2 ratio of 1:2 (two numbers) and 2:1. Out of which, in the first trimeric complex, a type-I NOD1-RIP2 heterodimer was found interacting with an RIP2^CARD^ using their type-IIa and IIIa interfaces. However, in the second and third heterotrimer, we observed type-I homodimers of NOD1 and RIP2 CARDs were interacting individually with RIP2^CARD^ and NOD1^CARD^ (in type-II and type-III interface), respectively. Overall, this study provides structural and dynamic insights into the NOD1-RIP2 oligomer formation, which will be crucial in understanding the molecular basis of NOD1-mediated CARD-CARD interaction in higher and lower eukaryotes.

## Introduction

Innate immunity plays a crucial role in host-defence mechanism against infectious pathogens, which is governed by a set of germ-line encoded receptors, called as pattern recognition receptors (PRRs) [1]. PRRs are generally activated by invading pathogenic patterns; termed as pathogen-associated molecular patterns (PAMPs) and/or self-generated danger signals, called as damage/danger-associated molecular patterns (DAMPs) [2,3]. These PRRs are classified into five major groups on the basis of their sub-cellular location, domain architecture, structural fold, specificity to ligands and molecular function. Among the five major groups; *i*.*e*., Toll-like receptors (TLRs) and C-type lectin receptors (CLRs) are membrane bound, whereas, NOD-like receptors (NLRs), RIG-I-like receptors (RLRs) and AIM2-like receptors (ALRs) are intra-cytoplasmic in nature [4].

Till date, a total of 22 numbers of NLRs have been reported in human and are grouped into five different sub-families based on their N-terminal domain distributions *i*.*e*., NLRA, NLRB, NLRC, NLRP and NLRX [5]. The NLR family members show tripartite domain architecture and are characterized by the presence of central nucleotide-binding and oligomerization domain (NOD/NACHT; found in NAIP, CIITA, HET-E and TP1 proteins). In addition to NACHT, they also contain one/two effector-binding domain/s [EBD: CAspase Recruitment Domain (CARD)/ PYrin Domain (PYD)] towards the N-terminal end and a varied number of Leucine Rich Repeats (LRRs) towards the C-terminal region [6–8].

NOD1 is one of the well-characterized receptors in NLRC sub-group and plays a pivotal role in host-defence mechanism. It is activated by recognizing iE-DAP (γ-ᴅ-Glu-meso-diaminopamelic acid, a bacterial peptidoglycan (PGN)), which in turn, triggers nuclear factor-kappa B (NF-κB) signalling [9]. The ligand recognition leads to the activation of the receptor and that causes its conformational alteration, where LRR unfolds from NACHT. Subsequently, the NACHT domain self-oligomerize in ATP-dependent fashion (an unexplored hypothesis), which aid in the transmission of signal(s) to adaptor molecule RIP2 (receptor-interacting serine/threonine protein kinase 2), through CARD-CARD interaction for the subsequent activation of NF-κB signalling cascade and maturation and release of pro-inflammatory cytokines [10]. The dysregulation of *NOD1* gene is associated with a number of inflammatory diseases. It has been noticed that certain SNPs (single nucleotide polymorphisms) in *NOD1* gene are associated with inflammatory bowel’s disease (IBD), eczema and atopic asthma [11, 12]. For instances, the non-synonymous variant rs2075820 (E266K) in *NOD1* gene is associated with increased risk to peptic ulcer patients upon *Helicobacter pylori* infection [13] and rs2709800 is associated with gastric lesions [14].

The well-studied interaction partner of NOD1 (and NOD2) is RIP2, which helps in transmitting the danger signal(s) for the activation of NF-κB and MAPK signalling pathway. Over last decade, significant efforts have been made by several groups for the exploration of the exact binding patches/interfaces responsible for NOD1- (even NOD2-) mediated CARD-CARD interaction [15–20]. Multiple groups have proposed multiple modes of interactions that include; surface charge interaction, acidic-basic surface interaction and multiple interface interactions [15–20]. However, the elucidation of the interaction modes and binding interfaces of CARDs (of NOD1 and RIP2) has still remained inconclusive. Therefore, in this study, an attempt has been made through a combinational approach involving protein-protein docking followed by multiple molecular dynamics (MD) simulation to delineate the most probable interaction modes, binding interfaces and critical residues involved in NOD1-mediated CARD-CARD interaction. Overall, this study will help in understanding the structural and dynamic features of CARD-CARD interaction, which is expected to stimulate the NOD1-mediated signalling mechanism in a broader perspective.

## Computational Methods

### Data retrieval and calculation of electrostatic surface potential

The amino acid sequences of NOD1 (NP_006083) and RIP2 (NP_003812) were retrieved from NCBI protein database and the experimentally solved 3D structures of NOD1^CARD^ (PDB ID: 2DBD) and RIP2^CARD^ (2N7Z) [21] were retrieved from PDB (https://www.rcsb.org). The sequence alignments were performed using ClustalX [22]. The binding sites were obtained from APAF1-CASP9 (Ap1-C9) CARD-CARD interfaces (PDB ID: 3YGS [23], 4RHW [24]) and NOD1-RIP2 [15, 19] interaction studies. The electrostatic surface potential of NOD1 and RIP2 CARDs were calculated using Adaptive Poisson-Boltzmann Solver (APBS) [25] with a grid spacing of 0.4 Å, salt concentration of 0.15 M and temperature of 296 K. The dielectric constants were set to ε = 2 for protein and ε = 78 for the solvent.

### Modelling of dimeric and trimeric complexes

In order to obtain the possible interaction modes governed by CARDs of NOD1 and RIP2, we performed several docking calculations using PyMOL (superimposition protocol). Primarily, two CARD-CARD complexes were created in reference to Ap1-C9 heterodimer complex (3YGS [23]); where, in complex-I, we superimposed NOD1^CARD^ with Ap1^CARD^ and RIP2^CARD^ with C9^CARD^ (where helix-α1 and α4 of RIP2^CARD^ (basic surface; type-Ia) were found facing to the helix-α2 and α3 (acidic surface; type-Ib) of NOD1^CARD^); however in complex-II, both NOD1 and RIP2 CARDs were superimposed with C9 and Ap1 CARDs, respectively (*i*.*e*., in reverse order of complex-I) (S1A and S1B Fig). Secondly, to perceive the type-II and III interaction interfaces, we docked a monomer (either NOD1 or, RIP2 CARD) (considering the Ap1-C9 heterotrimer complex (S1C Fig); 4RHW [24]) in the third position of stable complex that resulted from MD simulation (see ‘Results and Discussion‘ section) (S1D and S1E Fig). Here, in the trimeric complexes, NOD1 and RIP2 CARDs were superimposed with C9 and Ap1 CARDs in first and second position and in the third position, either an NOD1 and/or RIP2^CARD^ was superimposed with second Ap1^CARD^ (S1F Fig). In addition, we also modelled two type-I homodimers of NOD1^CARD^ and RIP2^CARD^ in reference to CASP1^CARD^ type-I dimeric conformation (5FNA) [26].

### Molecular dynamics simulation

In order to explore the dynamic features of individual CARDs of NOD1 and RIP2, and their modelled complexes; MD simulations were performed in GROMACS 4.5.5 [27] simulation suite using Amber99sb-ILDN force field [28] in periodic boundary conditions. The individual CARD structures and modelled complexes were solvated with TIP3P water models within individual cubic boxes. A minimum distance of 10-12Å was retained between protein surfaces and box-edge. Each simulation system was neutralized by adding a physiological strength (0.15M) of counter ions (Na^+^ and Cl^-^). Steepest descent energy minimization was performed until a tolerance of 1000 kJ mol^-1^ to remove steric conflicts between atoms and to avoid high-energy interaction. The energy minimized systems were position-restrained for 1 nanosecond (ns) in two different phases’ *viz*., NVT (constant volume) and NPT (constant temperature). The final production runs for individual CARDs and docked complexes were carried out for 50ns and 60ns for type-I dimeric and trimeric complexes, respectively. In this study, for each complex [heterodimeric and -trimeric (NOD1-RIP2-RIP2: complex-I and NOD1-RIP2-RIP2: complex-II)], three independent production runs were performed for better sampling of results. Moreover, MD simulation of homodimers (of NOD1/RIP2 CARDs) was performed for 50ns to delineate the existence/stability of type-I homodimer. The atomic compositions of all simulation systems were summarised in S1 Table.

### Analysis of MD trajectories

The trajectory analysis was performed using the integrated modules of GROMACS and VMD 1.9.2 [29]. To gauge the dynamic stability, the backbone root mean square deviation (RMSD) and radius of gyration (Rg) of individual CARDs, as well as CARD-CARD complexes, were analysed using *g_rmsdist* and *g_gyrate* tools, respectively. The *g_hbond* tool was used to calculate the total numbers H-bonds that formed between counter CARDs during the course of simulation time. The most widely used method, principal component analysis (PCA) was performed to observe the most relevant and/or correlated motions from the MD trajectories [30], by creating the covariance matrix of main chain atoms of the complexes using *g_covar* tool. To understand the global motion of CARD complexes (either by structurally or, in phase space), we analysed and projected the fast two principal components (PC1 and PC2) using *g_anaieg* tool. The *g_dist* and *g_mindist* tools were used for calculating interatomic and inter-residual dynamic distances of the intermolecular interactions; those formed between CARD counterparts during the course of the simulation. Molecular visualizations and interaction analysis were performed using PyMOL (academic license) and DIMPLOT (integrated in LigPlot+ v1.4.5; academic license) [31, 32]. The 2D graphs were generated by Grace 5.1.25 program (http://plasma-gate.weizmann.ac.il/Grace/).

## Results and Discussion

### Structural stability and electrostatic surface potential of individual CARDs

**Fig 1:**
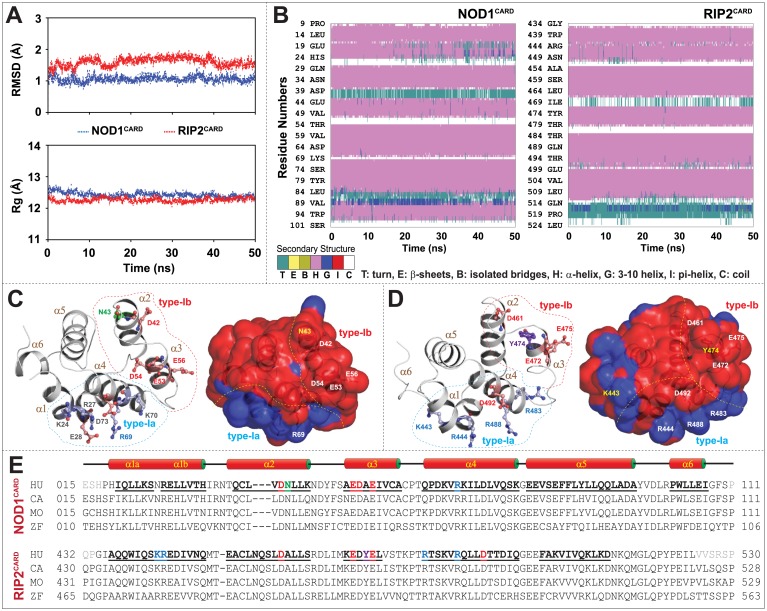
Structural stability, overview of individual CARDs and the key residues that drive CARD-CARD interaction. (A) Graphs depict the backbone RMSD and Rg of individual CARDs. (B) Secondary structure components of both NOD1 and RIP2 CARDs during the course of simulation time (the colour segments provided just below the figure represents the secondary structure properties). (C) Cartoon representations of NOD1^CARD^ and (D) RIP2^CARD^ displayed the six-helix structural folds (left panel) and electrostatic surface potentials (right panel). The key residues (that were reported or, predicted (in this study) to be involved in CARD-CARD interaction; were presented in sphere-stick model in different colours. The predicted residues are presented in grey and reported residues were portrayed according to their physicochemical properties. The surface view of CARD models shows distinct positive (blue) and negative (red) surface patches. The dotted lines represent the type-I surface patches (blue: type-Ia and red: type-Ib). (E) Multiple sequence alignment of NOD1 and RIP2 CARDs from human (HU), cattle (CA), mouse (MO) and zebrafish (ZF) indicates the key interacting residues. The reported key interacting residues are coloured based on their physicochemical properties.

The dynamic stability and compactness of CARDs were gauged by calculating the backbone RMSD and Rg from the MD trajectories. The backbone RMSD and Rg were found to be stable in both the CARDs as displayed in Fig 1A. The solved NOD1^CARD^ (2DBD) showed six numbers of closely packed α-helices; however, in RIP2^CARD^ (2N7Z) 6^th^ helix position was proposed to be a coil [21]. The analysis of secondary structure from MD trajectory seemed quite stable during the simulation period and a 3_10_-helix cum turn was noticed in the α6 position of RIP2^CARD^ (Fig 1B). Though the structural fold of both CARDs was found to be alike, the calculated electrostatic surface potential exhibited differential surface charge distribution. NOD1^CARD^ showed an extensive negative surface and a small positively charged patch; however, RIP2^CARD^ portrayed several positive and negative patches over the surface (Fig 1B and 1C). Owing to the interest of this study, two potential type-I surface patches (both positive (α1 and α4; type-Ia) and negative (α2 and α3; type-Ib)) were identified on the basis of earlier reports on NOD1/NOD2-RIP2 and Ap1-C9 CARD-CARD interaction [15–20,23,24,33,34]. The binding patches including the critical/surface exposed residues (experimentally proved/predicted) were illustrated in Fig 1C–1E.

### Interaction types, critical residues and molecular docking

The electrostatic component is the basis of CARD-CARD interaction. An observation based on Ap1-C9 complex (3YGS and 4RHW) solicits that acidic surface (helix-α2 and α3; type-Ib) of Ap1^CARD^ and basic surface (α1 and α4; type-Ia) of C9^CARD^ are indispensable type-I interface [23,24]. However, a recent observation revealed that type-I and type-II interfaces were necessary for Ap1-C9 interaction (4RHW) and a type-III interaction mode was noticed in the same complex between two Ap1^CARD^, which is crucial for apoptosome activity [24]. So far, Ap1-C9 (4RHW) and RIG-I-MAVS (4P4H) are two solved heterotrimeric CARD-CARD complexes, which clearly describe all three interface types (type-I, II, and III) [24,35].

Over the last decade, different mutational studies and the sequence-structure analysis has revealed that two possible binding patches *i*.*e*., either basic (α1 and α4) and/or acidic (α2 and α3) are crucial for NOD1/2-RIP2-mediated CARD-CARD interaction [15–18]. According to Manon *et al*. [15] an acidic surface (E53, D54, and E56) of NOD1^CARD^ and basic surface (R444, R483, and R488) of RIP2^CARD^ were essential for NOD1-RIP2 interaction, and the findings were well supported by Boyle and co-workers [18]. Conversely, in the same experiment [15], it was also reported that ‘R69E’ mutation (from basic surface) reduced the interaction ability of NOD1^CARD^ with RIP2^CARD^. In contrast, Fridh and Rittinger [17] proposed that the acidic surface of RIP2^CARD^ (D461, E472, E475, and D492) might be essential for NOD1-RIP2 interaction. However, a recent report on NOD1-RIP2 interaction proposed that a single interface (either basic or, acidic surface of NOD1^CARD^/RIP2^CARD^) might be insufficient for signal transmission [19]. Therefore, a total of four CARD-CARD complexes were prepared (S1 Fig; see ‘Computational Methods‘ section) and optimized through MD simulation.

### Type-Ia interface of NOD1^CARD^ is essential for 1:1 interaction

#### Analysis of complex stability

**Fig 2:**
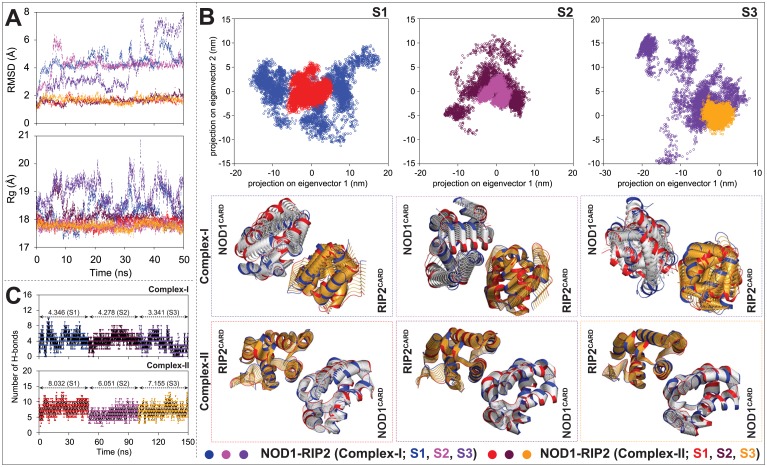
Analysis of complex stability of heterodimers and total numbers of intermolecular H-bonds formed during the course of simulation. (A) Graphs represent the backbone RMSD and Rg of NOD1-RIP2 CARD-CARD heterodimeric complexes (analysed from three individual trajectories (S1, S2, and S3)). (B) Principal component analysis of the said complexes. The projection graphs of first two eigenvectors (x-axis represents the PC1 and y-axis symbolizes PC2) of individual CARD-CARD complexes (type-I mode) indicate the global motion of complexes in phase space (upper panel). In lower panel, the superimposition of structural coordinates associated with the principal component 1 (PC1) of NOD1-RIP2 complexes display the global motion. The initial conformations were colour in blue, the final in red and the intermediate ones were displayed in silver (NOD1^CARD^) and orange (RIP2^CARD^). (C) The graphs indicate total number of H-bonds that formed by individual CARD-CARD complexes (type-I mode) during 50ns MD simulation in all three individual production runs.

In order to identify the probable NOD1-RIP2 CARD-CARD dimer, we inferred the stability and compactness of the complexes by calculating the backbone RMSD and Rg; and performed PCA to detect the list-motioned CARD-CARD complexes. The RMSD and Rg graphs from three individual trajectories (S1, S2, and S3) retained a stable backbone deviation and compactness in complex-II; however, in complex-I, unstable backbone deviation, and gyradius were noticed (Fig 2A). Similarly, the comparative PCA results revealed that complex-I showed a higher eigenvalue and occupied a larger area in phase space; whereas, complex-II covered a smaller area and showed distinct clusters (Fig 2B). Moreover, the visual inspection to the dynamic motion of the complexes indicated a higher motion in complex-I than in complex II (Fig 2B).

#### H-bond analysis

H-bonding is one of the major intermolecular forces that govern the binding stability of the protein and its partner(s). For the better understanding of the binding stability and nature of interaction, we analysed the intermolecular H-bonds (between CARDs of NOD1 and RIP2) of heterodimeric complexes as a function of simulation time. The analysed H-bonds (from all three individual simulations; S1, S2, and S3) in complex-II showed higher numbers of H-bonds and retained a stable H-bonding pattern; however, an unstable pattern and lesser numbers of H-bonds were noticed in complex-I (Fig 2C). Altogether, considering the above observations into account, the complex-II was projected as stable 1:1 NOD1-RIP2 complex for interaction study.

#### Dynamic interaction analysis (NOD1^CARD^-RIP2^CARD^ type-I interface)

**Fig 3:**
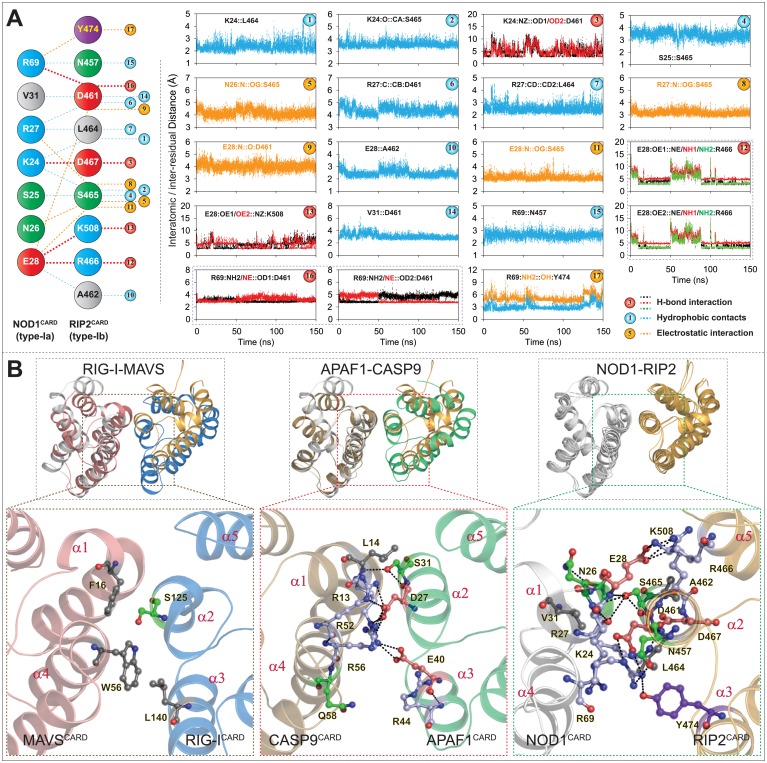
Illustration of intermolecular interactions governed by NOD1 and RIP2 CARDs. (A) All possible intermolecular interactions governed by type-Ia interface of NOD1^CARD^ and type-Ib interface of RIP2^CARD^ (acquired from S2 Fig) are presented in left panel and the corresponding dynamic distances (both atomic and residual) of the mentioned interacting pairs are outlined in right panel. (B) Structure comparison of NOD1-RIP2 CARD-CARD complex with solved type-I heterodimers; left panel: with RIG-I-MAVS (4P4H); middle: Ap1-C9 CARD-CARD complex (3YGS); right panel: superimposed view of final snapshots of NOD1-RIP2 CARD-CARD complexes (obtained from three individual production runs). The detailed type-I mode of molecular interactions of respective complexes were presented in lower panel. The critical interacting residues (involved in respective CARD-CARD interactions; experimental/ predicted (this study)) were potrayed in ball-stick model and the residues were coloured based on their physicochemical properties.

The results from Manon and co-workers have revealed that E53, D54, and E56 of NOD1^CARD^ (acidic patch) and R444, R483 and R488 (basic patch of RIP2^CARD^) are crucial for NOD1-mediated CARD-CARD interaction; however, in the same experiment, the group proposed a charge reversal mutation (R69E in NOD1^CARD^) abrogated the downstream signalling [15]. In another study, Fridh and Rittinger [17] proposed that the mutations, D461A, E472A, E475A and D492A (acidic patch of RIP2^CARD^) disrupt NOD1-RIP2 interaction. Moreover, Mayle *et al*. anticipated the critical role of Y474 (of RIP2^CARD^ type-Ib patch) in NOD1-mediated CARD-CARD interaction [19]. In the present study, the final snapshots from all three stable individual productions runs (S1, S2, and S3 of complex-II) were considered as representative structures to perceive the detailed intermolecular interactions between NOD1^CARD^ and RIP2^CARD^. The interaction analysis of all three individual snapshots (observed by DIMPLOT [31]) showed an average of 7–8 numbers of H-bonds (S2 Fig). The residues, K24, E28 and R69 of NOD1^CARD^, and D461, R466, D467 and K508 of RIP2^CARD^ were found to be involved in H-bonding. In addition to H-bonds, all other possible interactions were considered for dynamic interaction calculation as shown in Fig 3A. Further, to observe the consistency and stability in intermolecular interactions driven by NOD1 and RIP2 CARDs, the interatomic and inter-residual distances were calculated. The distance calculation result revealed six numbers of dynamic H-bonds (which include two strong salt bridges, R69-D461 and E28-R466 and two H-bonds, K24-D461, and E28-K508) (Fig 3A; left panel). In addition to H-bonds, we also noticed five electrostatic interactions (N26-S465; R27-S465; E28-D461; E28-S465; R69-Y474) and eight hydrophobic contacts between K24, S25, R27, E28, V31 and R69 (of NOD1^CARD^), and N457, D461, A462 and S465 (of RIP2^CARD^) (Fig 3A; right panel). However, the interactions of E472, E475 and D492 (of RIP2^CARD^; proposed by Fridh and Rittinger [17] couldn’t be established in type-I interface due to altered side chain orientation and position. The presented mode of NOD1-RIP2 CARD-CARD interaction model was found be similar with that of type-I mode of interaction as reported earlier in heterodimeric CARD complexes (Ap1-C9 [23, 24] and RIG-I-MAVS [35]; (Fig 3B)). A close visual inspection to the type-I mode of NOD1-RIP2 interaction (Fig 3B; right panel) partially supports the finding of Manon *et al*. [15] (on R69’s involvement); Mayle *et al*. [19] (on the participation of type-Ia surface patch of NOD1^CARD^ and type-Ib interface including ‘Y474’ of RIP2^CARD^) and Fridh and Rittinger (on D461 of RIP2^CARD^) [17].

### Involvement of multiple interfaces in NOD1-RIP2 interaction

The experimentally solved oligomeric CARD complexes portrayed three different types of interaction, *i*.*e*., type-I, II, and III [24, 35]. In a recent study, Mayle *et al*. suggested that a single interface interaction might be insufficient for signal transduction and proposed the existence of type-I and type-III interface [19]. Additionally, the authors denied the participation of type-Ia interface of RIP2^CARD^ and type-Ib interface of NOD1^CARD^ in NOD1:RIP2 heterodimer formation [19]. Here, we observed that the type-Ia interface of NOD1^CARD^ and type-Ib interface of RIP2^CARD^ might be a suitable interface for NOD1:RIP2 1:1 CARD-CARD interaction, which is consistent to Mayle and co-author’s observation [19]. To perceive the possibility of type-II and III mode of interaction, we modelled two trimeric complexes (complex-I: NOD1-RIP2-NOD1; complex-II: NOD1-RIP2-RIP2) considering Ap1-C9 heterotrimer (4RHW [24]) as the template (see ‘Computational Methods‘ section; S1D–S1F Fig). Further, to determine the dynamic stability of the generated heterotrimeric CARD complexes, MD simulation of individual complexes was performed for 60ns.

#### Analysis of dynamic stability of the complexes and intermolecular H-bonds

**Fig 4:**
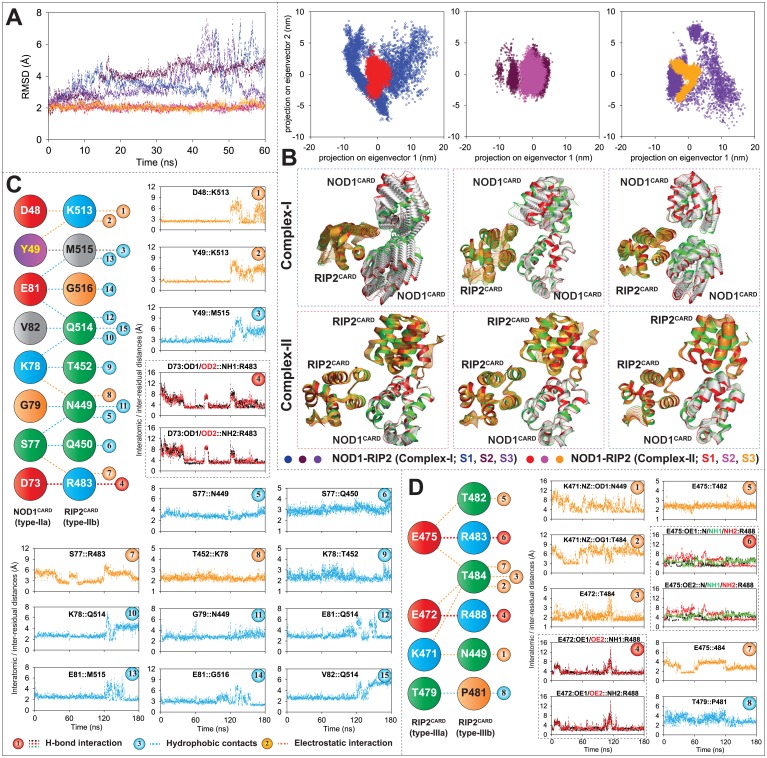
Complex stability and interatomic/inter-residual distance calculation. (A) Backbone RMSD of heterotrimeric (NOD1-RIP2-RIP2 and NOD1-RIP2-NOD1) CARD complexes. (B) Principal component analysis of heterotrimeric complexes (all three individual runs; S1, S2, and S3). Upper panel: 2D Projection of first two eigenvectors (PC1 and PC2) in phase space; lower panel: Superimposition of structural coordinates associated with first principal component (PC1). Initial confirmations were coloured in green and the finals were in red and the intermediate structures of NOD1 and RIP2 CARDs were illustrated in silver and orange, respectively. 2D representation of conserved interactions (acquired from S4A and S4B Fig) involved in type-II heterodimeric (C) and type-III RIP2-homorimeric interactions (D); the (atomic/residual) distance calculation graphs of all individual interaction were presented in the right panel of each diagram.

To understand complex stability, we have analysed the backbone RMSD of both the heterotrimeric complexes (complex-I and II) and performed PCA to observe the global motion. The calculated backbone RMSD of complex-II (NOD1-RIP2-RIP2) was found to be stable with a deviation of ~2.15 Å, whereas, an unstable and higher RMSD was perceived in complex-I (Fig 4A). Further, to obtain a list-motioned complex, we analysed the global motion of the complexes structurally as well as in phase space (by analysing the first two principal components: PC1 and PC2). As portrayed in Fig 4B, the scattered plots, and a comparatively larger motion was observed in complex-I; whereas in complex-II, we perceived distinct clusters in the phase space and list flexible complex structures.

Furthermore, to deduce the nature of binding stability, we calculated the total numbers of H-bonds between the monomers (in all three interfaces) as a function of simulation time. The H-bond analysis of type-I (NOD1-RIP2) and type-III interfaces (RIP2-NOD1; complex-I and RIP2-RIP2; complex-II) indicated the interaction stability (S3 Fig); however, in type-II interface, an unusual H-bonding pattern was noticed in complex-I (NOD1-NOD1) (S3A Fig). The superimposed complex structures (before and after MD) suggested a stable structural orientation in complex-II (S3B Fig). But in complex-I, the NOD1^CARD^ (in the third place) showed distorted orientations (S3A Fig), where it can be assumed that the type-II interfaces of NOD1 might be the unsuitable for homo-dimerization. Hence, complex-II (NOD1-RIP2-RIP2) was proposed as the stable heterotrimer.

#### Dynamic interaction analysis (of type-II and III interface)

To understand the intermolecular interactions governed by NOD1^CARD^ and RIP2^CARD^ in type-II interface and (between two) RIP2 CARDs in type-III interface, the final snapshot from each individual run (S1, S2, and S3) were considered as representative structures for interaction study. The interaction analysis of three snapshots was performed using DIMPLOT program (S4A and S4B Fig) and the conserved interaction pairs (Fig 4C and 4D) was taken for dynamic interaction analysis by calculating their interatomic/inter-residual distances. The interatomic/inter-residual distance graphs (of NOD1-RIP2 type-II interface) revealed ten hydrophobic contacts; four electrostatic interactions and two H-bonds (governed by D73 of NOD1^CARD^ and R483 of RIP2^CARD^ (Fig 4C)). Moreover, we also noticed some electrostatic interactions between D48, Y49, S77 and K78 of NOD1^CARD^ and T452, R483 and K513 of RIP2^CARD^ and several hydrophobic contacts between Y49, S77, K78, G79, E81 and V82 (of NOD1^CARD^) and N449, Q450, T452, Q514, M515, G516 (of RIP2^CARD^). Out of the interacting residues proposed for the type-II interface, only R483 was anticipated by Manon *et al*. [15] to be involved in the NOD1-RIP2 interaction. In type-III interface (between two RIP2 CARDs), the interaction was found to be dominated by H-bonds and electrostatic components (Fig 4D). The distance calculation revealed that E472 and E475 (of type-IIIa interface), and R483 and R488 of type-IIIb interface of RIP2^CARD^ were involved in H-bonding. Further, we also noticed that K471 and T479 (type-IIIa) formed electrostatic interaction and hydrophobic contacts with N449, P481, T482 and T484 (type-IIIb) (Fig 4D).

**Fig 5:**
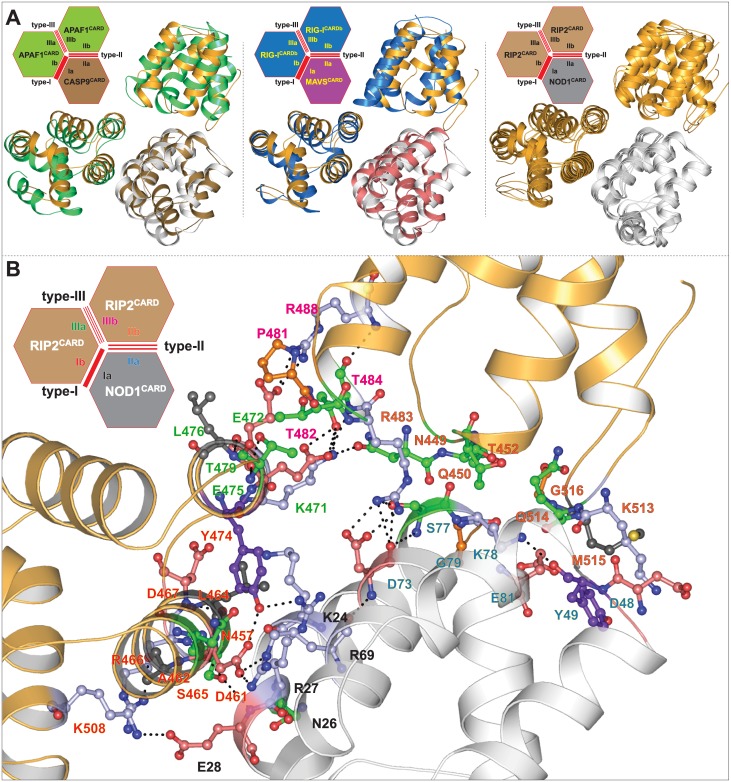
Heterotrimeric complex comparison and interaction analysis. (A) Comparison of proposed NOD1-RIP2 heterotrimeric complex (left panel: with Ap1-C9; middle: with RIG-I-MAVS; right panel: superimposed view of the said complexes taken from all three individual runs). (B) Illustration of detailed molecular interaction of NOD1-RIP2-RIP2 trimeric CARD complex. The critical amino acids involved in interactions were visualized in ball-stick model and are coloured according to physicochemical parameters. The interface residues were labelled in different colours (black: type-Ia; red: type-Ib; blue: type-IIa; orange: type-IIb; green: type-IIIa; magenta: type-IIIb). 2D representations of heterotrimeric complexes were visualized (in right-upper corner of each illustration) for better understanding of trimeric interface interactions.

The proposed model of NOD1-RIP2-RIP2 heterotrimer complex was found similar to that of Ap1-C9 [24] and RIG-I-MAVS [35] trimeric conformations (Fig 5A). As illustrated in Fig 5B, the detailed tri-faced (NOD1-RIP2-RIP2) mode of interaction revealed ‘inter-surface/patch’ interaction network. In type-I mode of interaction, the residues K24, N26, R27, E28 and R69 of type-Ia patch (α1 and α4) of NOD1 was found to interact with N457, A462, L464, S465, R466, Y474 and K508 (type-Ib patch) of RIP2^CARD^ (Figs 3B and 5B). Also, it was noticed that D73 of NOD1^CARD^ (despite being present in type-Ia interface) formed strong H-bonds with R483 (type-IIIb patch) of RIP2^CARD^ and the interactions were found dynamically stable in all three trajectories (Fig 4C). It can be speculated that the inter-patch interaction (D73-R483) might be playing some important role in the signal transmission which needs experimental validation in the near future.

### NOD1 and RIP2 CARDs form type-I homodimer in heterotrimeric conformation

Manon *et al*. [15] and Mayle *et al*. [19] suggested the possibility of type-I homodimer in RIP2^CARD^ and Ver Heul *et al*. [36] revealed the helix-swapped homodimer of NOD1 (S5A Fig). After analysing the type-I electrostatic interfaces of NOD1 and RIP2 CARDs, we assumed the existence of the plausible type-I homodimer in NOD1 and RIP2 CARDs. To infer the dynamic existence of type-I homodimers, we modelled NOD1 and RIP2 homodimeric CARD complexes (see ‘Computational Methods‘ section) (S5B Fig) and performed a 50ns MD simulation. The dynamic stability, compactness, and the nature of interactions were analysed by calculating the backbone RMSD, Rg and intermolecular H-bonds as a function of simulation time. The resulting RMSD and Rg of homodimeric complexes indicated a stable backbone deviation and gyradius after 35ns of simulation time (S5C Fig). H-bond analysis revealed a conserved bonding pattern in NOD1^CARD^ dimer; whereas in case of RIP2, an unstable bonding pattern and lesser H-bonds (2.422) were noticed (S5D Fig). The superimposition of pre and post-MD structures indicated a tilted orientation of dimers (S5E Fig). Henceforth, to perceive the existence of type-I homodimers in heterotrimeric complex; the monomers of RIP2 and NOD1 CARD were docked individually to modelled type-I homodimers of RIP2^CARD^ and NOD1^CARD^ in reference to Ap1-C9 heterotrimer, as a result two other heterotrimeric complexes were created (NOD1-NOD1-RIP2: complex-III and RIP2-RIP2-NOD1: complex-IV)(4RHW [24]; S6A Fig). Thereafter, the modelled complexes were subjected to 50ns MD simulation to delineate their complex stability. To perceive the dynamic stability and the nature of the interaction, we analysed the backbone RMSD and Rg, and calculated the intermolecular H-bonds in all the interfaces. In complex-III, we noticed a stable backbone deviation and gyradius; while in complex-IV, a higher RMSD was observed after 33ns (S6B Fig). The calculated H-bonds (as a function of simulation time) and structural superimposition of heterotrimers (pre and post-MD) indicated lower RMS (2.495Å) and stable H-bonding pattern in complex-III (S6C Fig); whereas in complex-IV, we observed a higher RMS (4.445Å) and fewer H-bonds in type-III interface after 40ns of MD (S6D Fig).

#### Interaction analysis (type-I interfaces)

**Fig 6:**
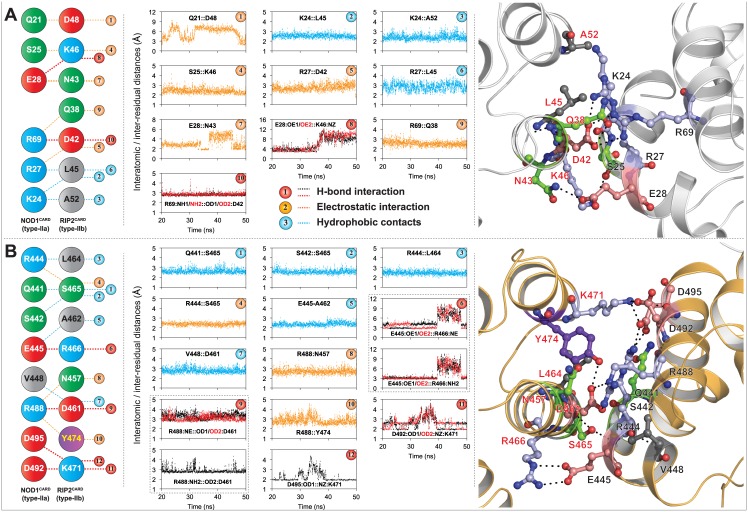
Illustration of homodimeric (type-I) mode of interaction. (A) NOD1^CARD^, (B) RIP2^CARD^; left panel shows the conserved interactions (acquired from S7A and S8A Figs, respectively) mediated by type-I interfaces of NOD1 and RIP2 CARDs, and the middle panel indicates respective inter-residual/interatomic distances. Detailed 3D representation type-I (1:1 homodimeric) interaction modes were depicted in the right panel. Critical residues involved in intermolecular interactions were shown in ball and stick model, and were coloured based on physicochemical parameters. The black dotted lines represent the inter/intra-molecular polar contacts. The labelled black and red fonts represent the type-Ia and Ib interface residues, respectively.

The interaction analysis of representative structures extracted from the trajectory (10ns interval) revealed a conserved interaction pattern in all the interfaces (S7 and S8 Figs). In the NOD1 homodimeric interface, we observed a strong salt bridge [between R69 (type-Ia) and D42 (type-Ib)], four electrostatic interactions (S25-K46, R27-D42, E28-N43, R69-Q38) and three hydrophobic contacts (K24-L45/A52, R27-L45) (Fig 6A). However, in RIP2 counterpart, we observed a total of four-five dynamically stable H-bonds (R488-D461, E445-R466, D492-K471), three electrostatic interactions (R444-S465, R488-N457/Y474) and five hydrophobic contacts (Q441/S442-S465, R444-L464, E445-A462, V448-D461) between the type-I interfaces of RIP2^CARDs^ (Fig 6B). Amongst the critical residues predicted to be involved in type-I homodimeric interactions (NOD1/RIP2; this study), D42, N43 and R69 of NOD1^CARD^ and R444, D461, Y478, R483, R488, D492 and D495 of RIP2^CARD^ are the residues that are already reported to be involved in NOD1/NOD2-RIP2 interactions [15, 17, 19].

### RIP2^CARD^ might be using its type-IIa interface for NOD1-RIP2 interaction

In first stable heterotrimeric complex (NOD1-RIP2-RIP2 (complex-II)), we observed a type-II mode of interaction between type-IIa interface of NOD1^CARD^ and type-IIb interface of RIP2^CARD^ (Fig 4C) and the similar type of interactions were also observed in complex-III (NOD1-NOD1-RIP2) (S7B Fig). However, in complex-IV (RIP2-RIP2-NOD1), though we observed stable interactions between the type-IIa and type-IIb interfaces of RIP2 and NOD1 CARDs (S8B Fig), which were found to be less in comparison to the type-II mode of interaction, which was observed in the first trimeric complex (NOD1-RIP2-RIP2) (Fig 4C). The second type-II interaction comprised of four H-bonds (including a salt bridge between D492 and R35), one electrostatic interaction (E472-N36) and five hydrophobic contacts (S9 Fig). Additionally, another salt bridge, which was being observed between R35 of NOD1^CARD^ (type-IIb) and E475 (type-IIIa) of second RIP2^CARD^ (S9 Fig; right panel) might be crucial for NOD1-RIP2 signal transmission mechanism.

### Type-III interfaces of both the CARDs are dynamically suitable for the interaction

**Fig 7:**
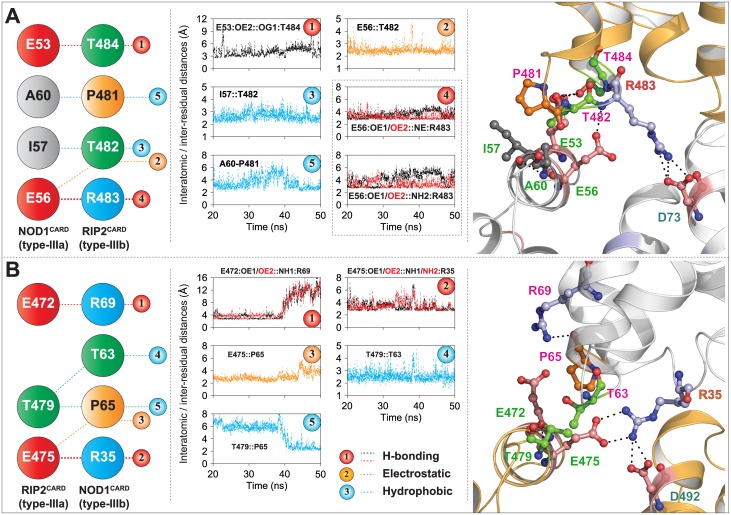
Type-III mode of NOD1-RIP2 interaction. (A) First type-III interface interaction mode, where type-IIIa interface of NOD1^CARD^ is found interacting with type-IIIb patch of RIP2^CARD^; (B) Second type-III mode of interaction (type-IIIa patch of RIP2^CARD^ interacts with type-IIIb patch of NOD1^CARD^). Left panel of both the figures shows the conserved interactions (taken from S7C and S8C Figs), which are mediated by type-III interfaces of NOD1 and RIP2 CARDs; middle panel displays respective inter-residual/interatomic distance graphs. The detailed type-III modes of interactions (along with inter-interface contacts) were depicted in the right panel. The critical residues involved in intermolecular interactions were portrayed in ball and stick model, and were coloured based on physicochemical parameters. NOD1 and RIP2 CARDs models were visualized in white and orange cartoon for better understanding. The black dotted lines represent the inter/intra-molecular polar contacts. The residues labelled in different colour fonts symbolizes the interface types (blue: type-IIa; orange: type-IIb; green: type-IIIa; magenta: type-IIIb).

In 2014, Mayle *et al*. explored the existence of type-III mode of interaction between NOD1 and RIP2 CARDs and proposed that E53 of NOD1^CARD^ (type-IIIa interface) and R483 (type-IIIb interface) of RIP2^CARD^ are crucial for interaction. In the current study, we observed two type-III modes of interactions between NOD1 and RIP2 CARDs. In complex-III, the interactions were noticed between the type-IIIa interface (E53, E56, I57, and A60) of NOD1^CARD^ and the type-IIIb patch (P481, T482, R483, and T484) of RIP2^CARD^ (Fig 7A); however in complex-IV, the crucial residues, E472, E475 and T479 (of the type-IIIa of RIP2^CARD^) and T63, P65 and R69 (type-IIIb patch of NOD1^CARD^) showed the interaction (Fig 7B). In addition, an inter-patch interaction (which includes two strong salt bridges) was noticed between ‘R35’ of NOD1^CARD^ and E475 (type-IIIa patch) and D492 (type-Ia interface) of RIP2^CARD^ (S9 Fig, Fig 7B). Of the interacting residues involved in second type-III heterodimeric interface, D492 of RIP2^CARD^ was proposed to be interacting with NOD1 and NOD2 CARDs [17]. The observed residues participating in type-III interface-interactions (this study), were already reported to be trivial (and/or vice versa) [15, 17, 19]. According to Mayle *et al*. the mutations of solvent exposed residues, D446, V448, T484, S485, K486, Q497 and E522 of RIP2^CARD^ had no role in the interaction with NOD1^CARD^; however, we noticed the active participation of T484 and Q497 in type-III (Fig 7) and type-II (S9 Fig) interfaces, respectively.

**Fig 8:**
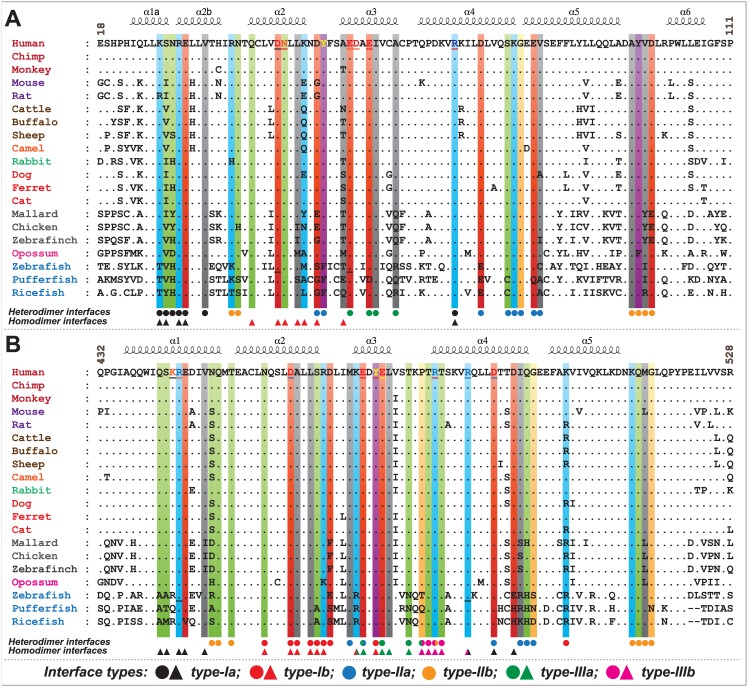
Multiple sequence alignment of NOD1 and RIP2 CARDs. Sequence alignment of (A) NOD1 and (B) RIP2 CARDs highlights the key residues that drive CARD-CARD interaction. The residues highlighted in different colour bars (based on physicochemical properties) were predicted to be involved in CARD-CARD interaction. The reported and/or predicted residues (from human and zebrafish) are underlined. Coloured circles and triangles (black: type-Ia; red: type-Ib; blue: type-IIa; orange: type-IIb; green: type-IIIa; magenta: type-IIIb) indicate the residues involved in hetero and homodimeric interactions, respectively. GenBank Accessions of NOD1 and RIP2 sequences were considered for multiple sequence analysis (MSA) are as follows: NOD1:- [Human (NP_006083), Chimp (XP_001165528), Monkey (XP_001085719), Mouse (NP_766317), Rat (NP_001102706), Cattle (NP_001243492), Buffalo (XP_006055719), Sheep (XP_011967635), Camel (XP_010986339), Rabbit (XP_002713781), Dog (XP_005628733), Ferret (XP_004762624), Cat (XP_011278751), Mallard (NP_001297310), Chicken (NP_001305367), Zebra finch (XP_012427146), Opossum (XP_001381520), Zebrafish (XP_002665106), Pufferfish (XP_003965935) and Ricefish (XP_011483244)]; RIP2:- [Human (NP_003812), Chimp (XP_519850), Monkey (XP_001084687), Mouse (NP_620402), Rat (NP_001178794), Cattle (NP_001029782), Buffalo (XP_006056644), Sheep (XP_011994115), Camel (XP_010976526), Rabbit (XP_008253935), Dog (XP_005638158), Ferret (XP_004767029), Cat (XP_011289616), Mallard (XP_005029607), Chicken (NP_001026114), Zebra finch (XP_002198519), Opossum (XP_016287297), Zebrafish (NP_919392), Pufferfish (XP_003975835) and Ricefish (XP_004078988)].

To understand the evolutionary perspective of critical residues involved in multimeric-interface interactions, we performed multiple sequence alignment of individual CARDs of NOD1 and RIP2 of various species and mapped the critical residues involved in intermolecular CARD-CARD interactions (Fig 8). As evident from multiple sequence alignment, the predicted interacting residues from homo/heterodimeric interfaces, R27, E28, V31, Q38, D42, N43, E53, I57, R69, K78, G79 and A96 of NOD1^CARD^ (Fig 8A) and R444, Q450, T452, N457, D461, A462, L464, M470, E472, Y474, E475, L476, T482, R483, T484, R488, D492, K513 and Q514 of RIP2^CARD^ (Fig 8B) were found to be conserved. However, a key residue ‘D73’ of NOD1^CARD^ [involved in inter-patch interaction in the NOD1-RIP2-RIP2 heterotrimeric complex (Fig 7B)] was found to be variable (replaced by ‘E’ in fish species). Particularly, some of the key charged residues, K24 of type-Ia, D48 (also involved in type-I homodimer) and E81 (replaced by ‘Q’ in pufferfish) of type-IIa, and E56 (replaced by ‘D’ in pufferfish) of type-IIIa interface of NOD1^CARD^, and R466 (replaced by ‘K’ in opossum), D467 and K508 (replaced by ‘R’) of type-Ib interface were found to be variable. Overall, the multiple sequence analysis revealed the existence of residual differences in higher and lower vertebrates, and the paradox in zebrafish [23] might be due to the differential structural orientation of critical type-I interface residues or the residual variation; which needs further experimentation for legitimacy.

## Conclusion

In the present study, we employed the structural bioinformatics approaches to irradiate the possible binding interfaces of NOD1 and RIP2 CARDs involved in the so-called CARD-CARD interaction. Our observation suggests the possible existence of NOD1:RIP2 type-I heterodimer and three heterotrimeric complexes with different combinations of NOD1 and RIP2 CARDs. Herein, we noticed that the alternately charged residue (E28 of type-Ia interface of NOD1^CARD^ and R466 of type-Ib interface of RIP2^CARD^) presumed to contribute to the interaction despite being present in charge reversal patch. Moreover, we detected an inter-patch/surface interaction between type-Ia interface residue ‘D73’ of NOD1^CARD^ and type-IIIb interface residue ‘R483’ of second RIP2^CARD^ in the first (NOD1-RIP2-RIP2) and second (NOD1-NOD1-RIP2) heterotrimeric complexes, which might be critical in transmitting danger signals for the propagation of NF-κB signalling. We also observed the type-I homodimeric existence of NOD1 and RIP2 CARDs in two dynamically stable heterotrimeric complexes, which can be considered as second and third trimeric complexes (probably) responsible for downstream signalling. Altogether, our study demonstrates the molecular basis of NOD1-mediated CARD-CARD interaction, which would open up better avenues to understand the binding interfaces that govern the said interaction. Finally, the amino acids predicted in this study to be involved in the intermolecular interaction warrants further investigation for the legitimacy of this work.

## Supporting Information

S1 Fig Illustration of docked heterodimeric and heterotrimeric complexes. Heterodimeric NOD1-RIP2 CARD-CARD complexes ((A) complex-I, (B) complex-II) and (C) APAF1-CASP9 dimer (3YGS); Heterotrimeric complexes of (D) NOD1-RIP2-RIP2 (complex-I); (E) NOD1-RIP2-RIP2 (complex-I); (F) APAF1-CASP9 (4RHW); 2D illustrations were displayed in the corner of each figure for better understanding of the docked complexes.
(TIF)

S2 Fig Figure displays the molecular interactions governed by CARDs NOD1 and RIP2. The molecular interactions of the final snapshots three individual simulations (of complex-II) were performed using DIMPLOT. The interacting residues are colored according to physicochemical parameters and the blue straight lines and orange dashed lines indicate the H-bonds and hydrophobic interactions, respectively.
(TIF)

S3 Fig The superimposed view of NOD1-RIP2 heterotrimeric complexes before and after simulation, and total numbers of intermolecular H-bonds governed by hetero/homodimeric interfaces of NOD1 and RIP2 CARDs, calculated from six individual trajectories. (A) NOD1-RIP2-NOD1 (complex-I); (B) NOD1-RIP2-RIP2 (complex-II). In superimposed cartoon structures, white cartoons represent the initial complex (pre-MD) and red (S1), green (S2) and blue (S3) indicate the post-MD complex structures. In the right-corner of each figure, 2D model of trimeric complex indicates the interaction types (the green tick mark designates the interaction possibility and the red cross-mark shows the unsuitable interfaces for interaction). Lower panel of each figure shows the total number of intermolecular H-bonds formed during the course of simulation time along three individual trajectories of two different complexes.
(TIF)

S4 Fig Detailed molecular interactions governed by NOD1 and RIP2 CARDs. (A) type-II and (B) type-III mode of interaction. The molecular interactions were performed using DIMPLOT. The interacting residues are colored according to physicochemical parameters and the blue straight lines and orange dashed lines indicate the H-bonds and hydrophobic interactions, respectively.
(TIF)

S5 Fig Structural and dynamic analysis of NOD1 and RIP2 CARD type-I homodimers. (A) Experimentally solved helix swapping model of NOD1; (B) manually docked type-I homodimer of NOD1^CARD^ (left panel) and RIP2^CARD^ (right panel). (C) The backbone RMSD and Rg of homodimeric complexes indicate the stable graphs in NOD1 homodimer. (D) Total number of H-bonds governed by homodimers during 50ns simulation time. (E) Superimposed cartoon view of homodimer structures before and after MD (homodimers of NOD1^CARD^ and RIP2^CARD^ dimers) indicate tilted orientations.
(TIF)

S6 Fig Heterotrimeric complex stability and H-bonds. (A) Modeled heterotrimeric complexes; [complex-III (NOD1-NOD1-RIP2): right panel], [complex-IV (RIP2-RIP2-NOD1): middle panel] and their superimposed view with APAF1-CASP9 heterotrimer (4RHW) (right panel). (B) Backbone RMSD and Rg of the trimeric complex. Superimposed cartoon view and the total numbers of H-bonds formed in three different interfaces (type-I, II, and III) of NOD1-RIP2 heterotrimeric complexes [(C) complex-III (NOD1-NOD1-RIP2), (D) complex-IV (RIP2-RIP2-NOD1)] during 50ns of simulation time.
(TIF)

S7 Fig 2D representation of all three types of interactions governed by NOD1 and RIP2 CARDs in complex-III (NOD1-NOD1-RIP2). (A) type-I (between two NOD1CARDs); (B) type-II (between NOD1 and RIP2 CARDs); and (C) type-III (between NOD1 and RIP2 CARDs). The molecular interactions were performed using DIMPLOT. The interacting residues are colored according to physicochemical parameters and the blue straight lines and orange dashed lines indicate the H-bonds and hydrophobic interactions, respectively.
(TIF)

S8 Fig Figure displays all three types interactions governed by NOD1 and RIP2 CARDs in complex-IV. (A) type-I (between two RIP2CARDs); (B) type-II (between RIP2 and NOD1 CARDs); and (C) type-III (between RIP2 and NOD1CARDs). The molecular interactions were performed using DIMPLOT. The interacting residues are colored according to physicochemical parameters and the blue straight lines and orange dashed lines indicate the H-bonds and hydrophobic interactions, respectively.
(TIF)

S9 Fig Detailed type-II mode interaction governed by CARDs of NOD1 and RIP2 in complex-IV. Left panel of the figure shows the conserved interactions (obtained from S9 Fig B) mediated by type-IIa interfaces of RIP2^CARD^ and type-IIb interface of NOD1^CARD^; the middle panel indicates respective inter-residual/interatomic distances and detailed 3D representation of type-II interaction mode was depicted in the right panel. Critical residues involved in intermolecular interactions were visualized in ball and stick model, and were colored based on physicochemical parameters. The black dotted lines represent the inter/intra-molecular polar contacts, and the labeled blue and orange fonts represent the type-IIa and IIb interface residues.
(TIF)

S1 Table Atomic compositions of all the simulation systems considered for molecular dynamics simulation. (DOC)
