# Supplementary figures and images for: NOD1CARD Might Be Using Multiple Interfaces for RIP2-Mediated CARD-CARD Interaction: Insights from Molecular Dynamics Simulation
Source: PLoS One. 2017 Jan 23;12(1):e0170232. doi: 10.1371/journal.pone.0170232 (PMC5256935; doi:10.1371/journal.pone.0170232)

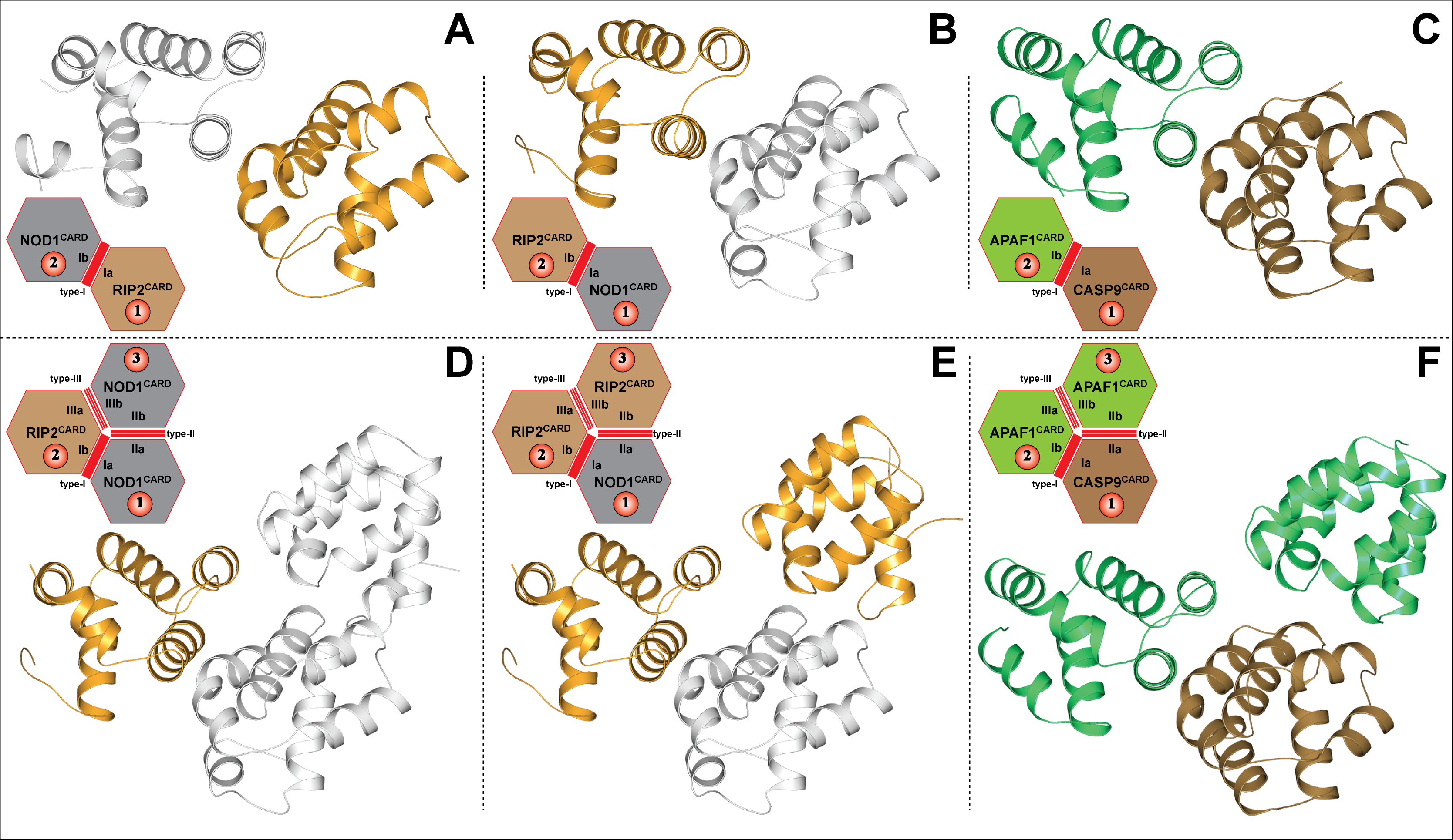

Supplement: S1 Fig — Heterodimeric NOD1-RIP2 CARD-CARD complexes ((A) complex-I, (B) complex-II) and (C) APAF1-CASP9 dimer (3YGS); Heterotrimeric complexes of (D) NOD1-RIP2-RIP2 (complex-I); (E) NOD1-RIP2-RIP2 (complex-I); (F) APAF1-CASP9 (4RHW); 2D illustrations were displayed in the corner of each figure for better understanding of the docked complexes. (TIF) [file pone.0170232.s001.tif]

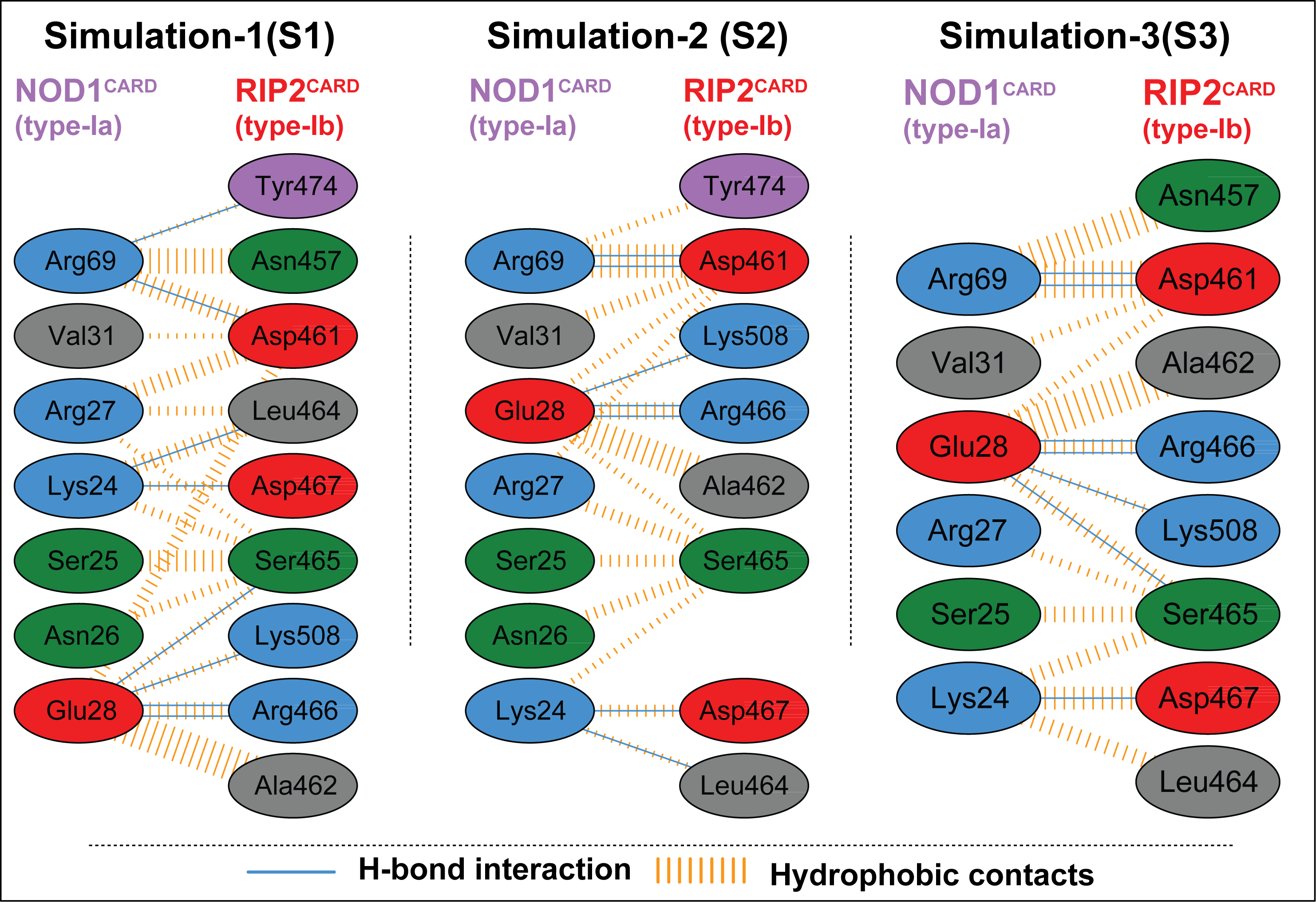

Supplement: S2 Fig — The molecular interactions of the final snapshots three individual simulations (of complex-II) were performed using DIMPLOT. The interacting residues are colored according to physicochemical parameters and the blue straight lines and orange dashed lines indicate the H-bonds and hydrophobic interactions, respectively. (TIF) [file pone.0170232.s002.tif]

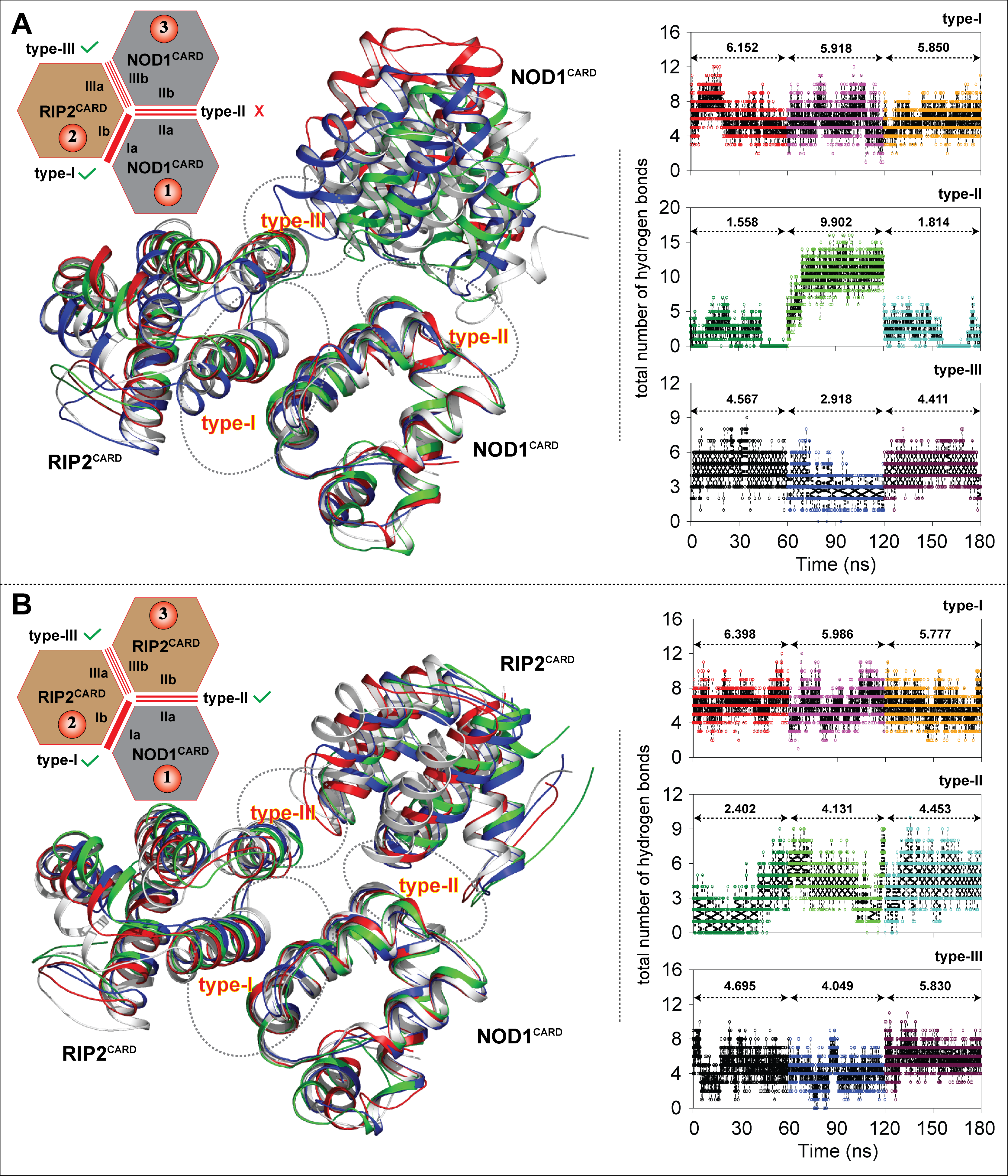

Supplement: S3 Fig — (A) NOD1-RIP2-NOD1 (complex-I); (B) NOD1-RIP2-RIP2 (complex-II). In superimposed cartoon structures, white cartoons represent the initial complex (pre-MD) and red (S1), green (S2) and blue (S3) indicate the post-MD complex structures. In the right-corner of each figure, 2D model of trimeric complex indicates the interaction types (the green tick mark designates the interaction possibility and the red cross-mark shows the unsuitable interfaces for interaction). Lower panel of each figure shows the total number of intermolecular H-bonds formed during the course of simulation time along three individual trajectories of two different complexes. (TIF) [file pone.0170232.s003.tif]

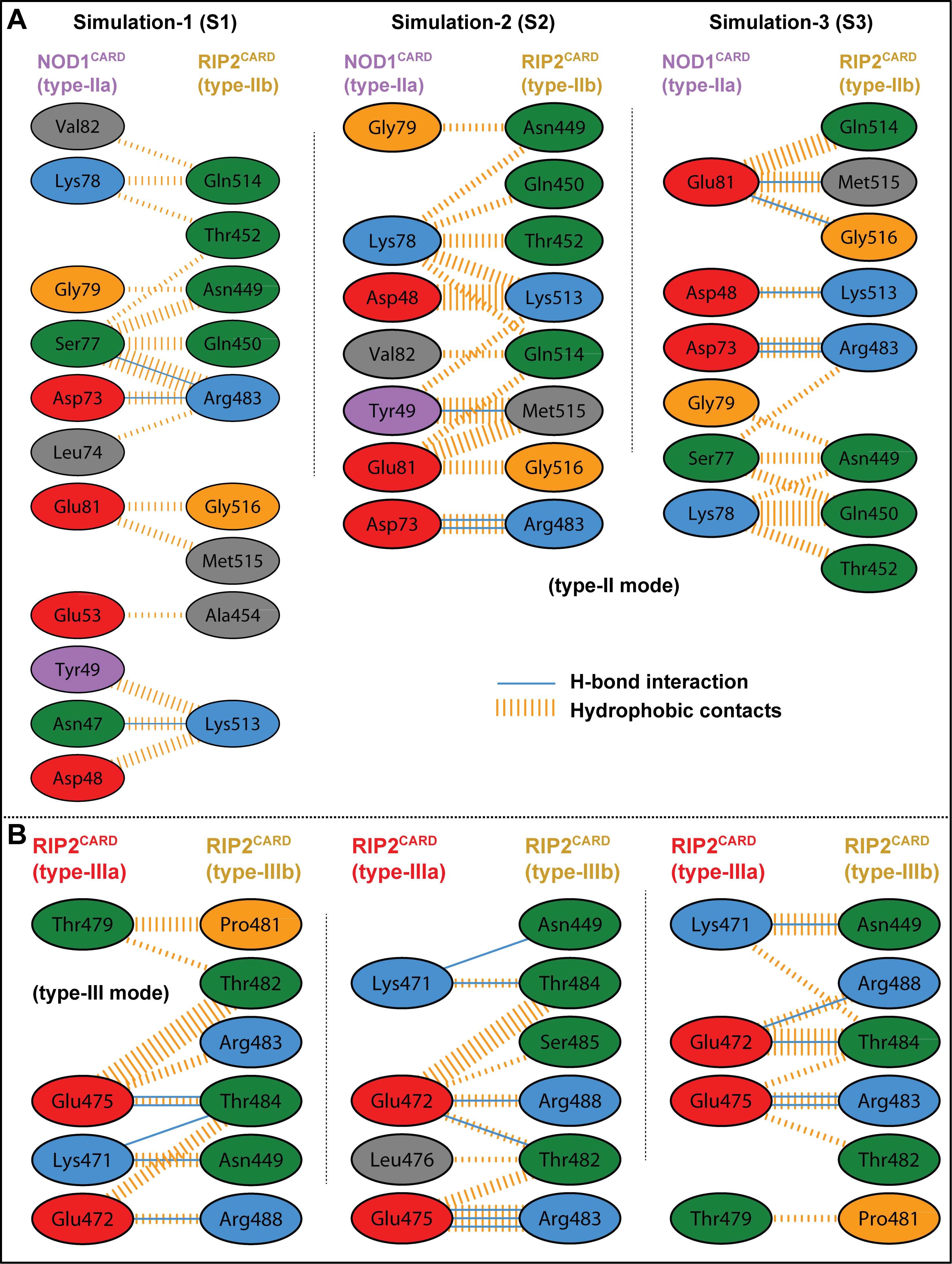

Supplement: S4 Fig — (A) type-II and (B) type-III mode of interaction. The molecular interactions were performed using DIMPLOT. The interacting residues are colored according to physicochemical parameters and the blue straight lines and orange dashed lines indicate the H-bonds and hydrophobic interactions, respectively. (TIF) [file pone.0170232.s004.tif]

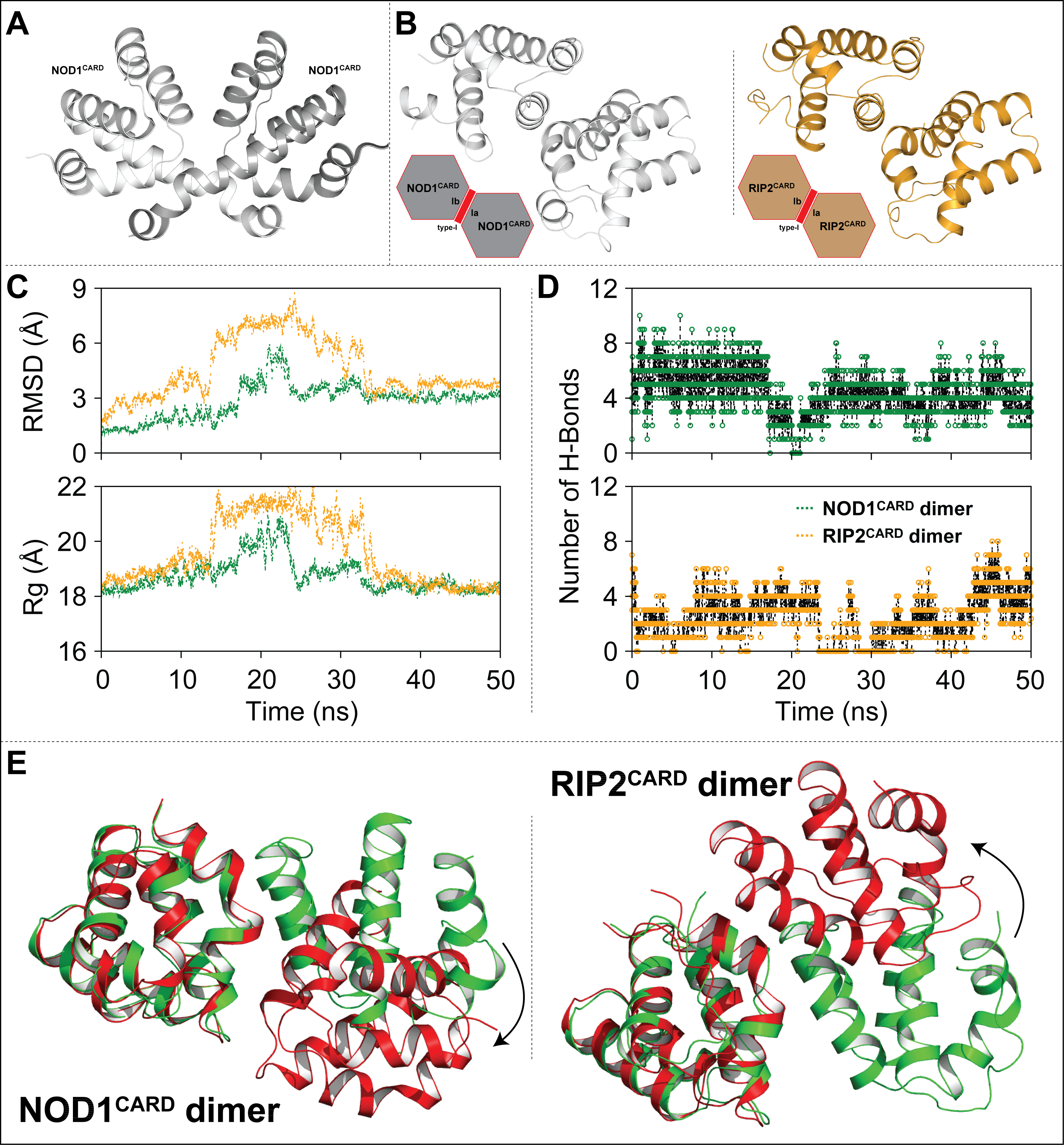

Supplement: S5 Fig — (A) Experimentally solved helix swapping model of NOD1; (B) manually docked type-I homodimer of NOD1CARD (left panel) and RIP2CARD (right panel). (C) The backbone RMSD and Rg of homodimeric complexes indicate the stable graphs in NOD1 homodimer. (D) Total number of H-bonds governed by homodimers during 50ns simulation time. (E) Superimposed cartoon view of homodimer structures before and after MD (homodimers of NOD1CARD and RIP2CARD dimers) indicate tilted orientations. (TIF) [file pone.0170232.s005.tif]

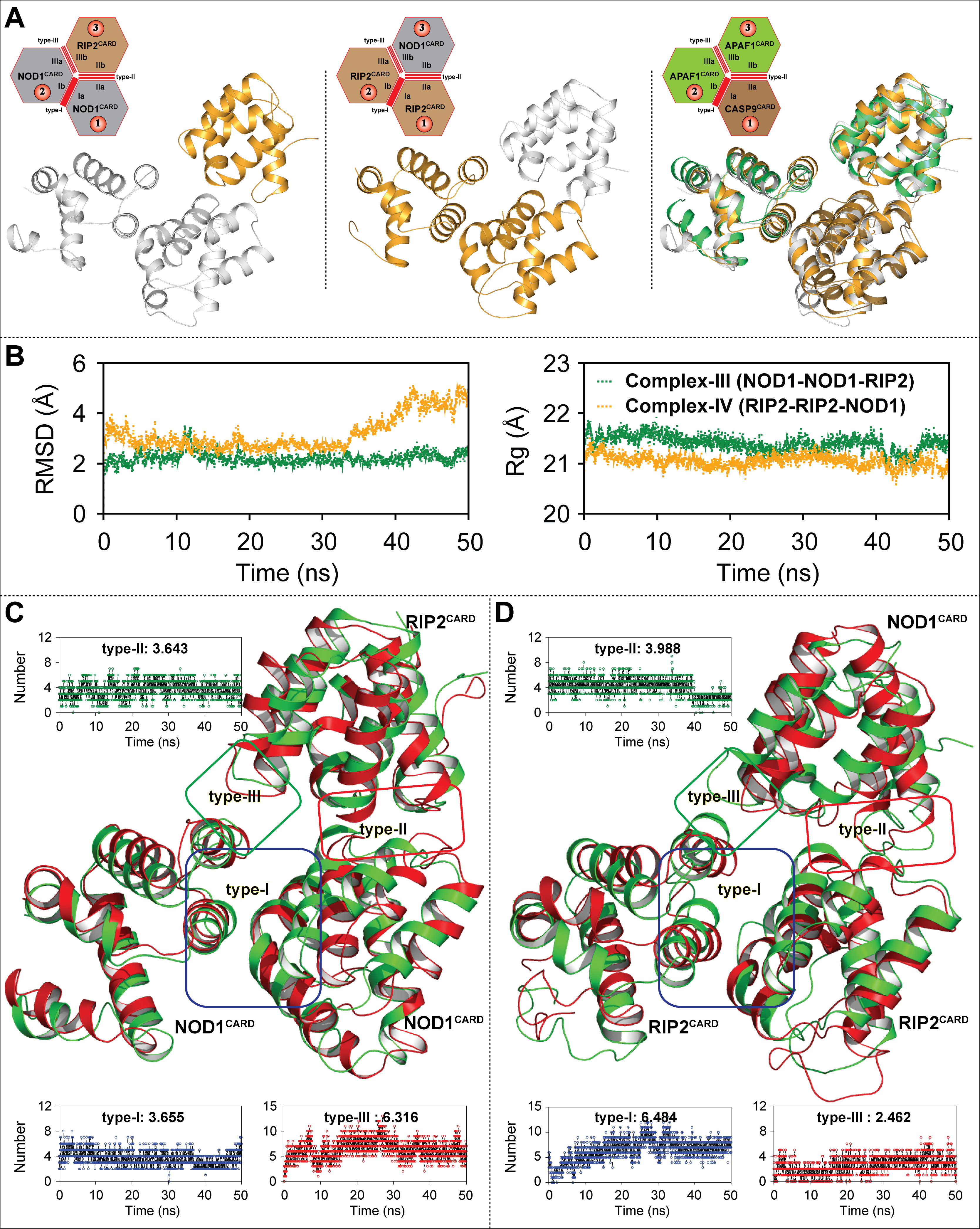

Supplement: S6 Fig — (A) Modeled heterotrimeric complexes; [complex-III (NOD1-NOD1-RIP2): right panel], [complex-IV (RIP2-RIP2-NOD1): middle panel] and their superimposed view with APAF1-CASP9 heterotrimer (4RHW) (right panel). (B) Backbone RMSD and Rg of the trimeric complex. Superimposed cartoon view and the total numbers of H-bonds formed in three different interfaces (type-I, II, and III) of NOD1-RIP2 heterotrimeric complexes [(C) complex-III (NOD1-NOD1-RIP2), (D) complex-IV (RIP2-RIP2-NOD1)] during 50ns of simulation time. (TIF) [file pone.0170232.s006.tif]

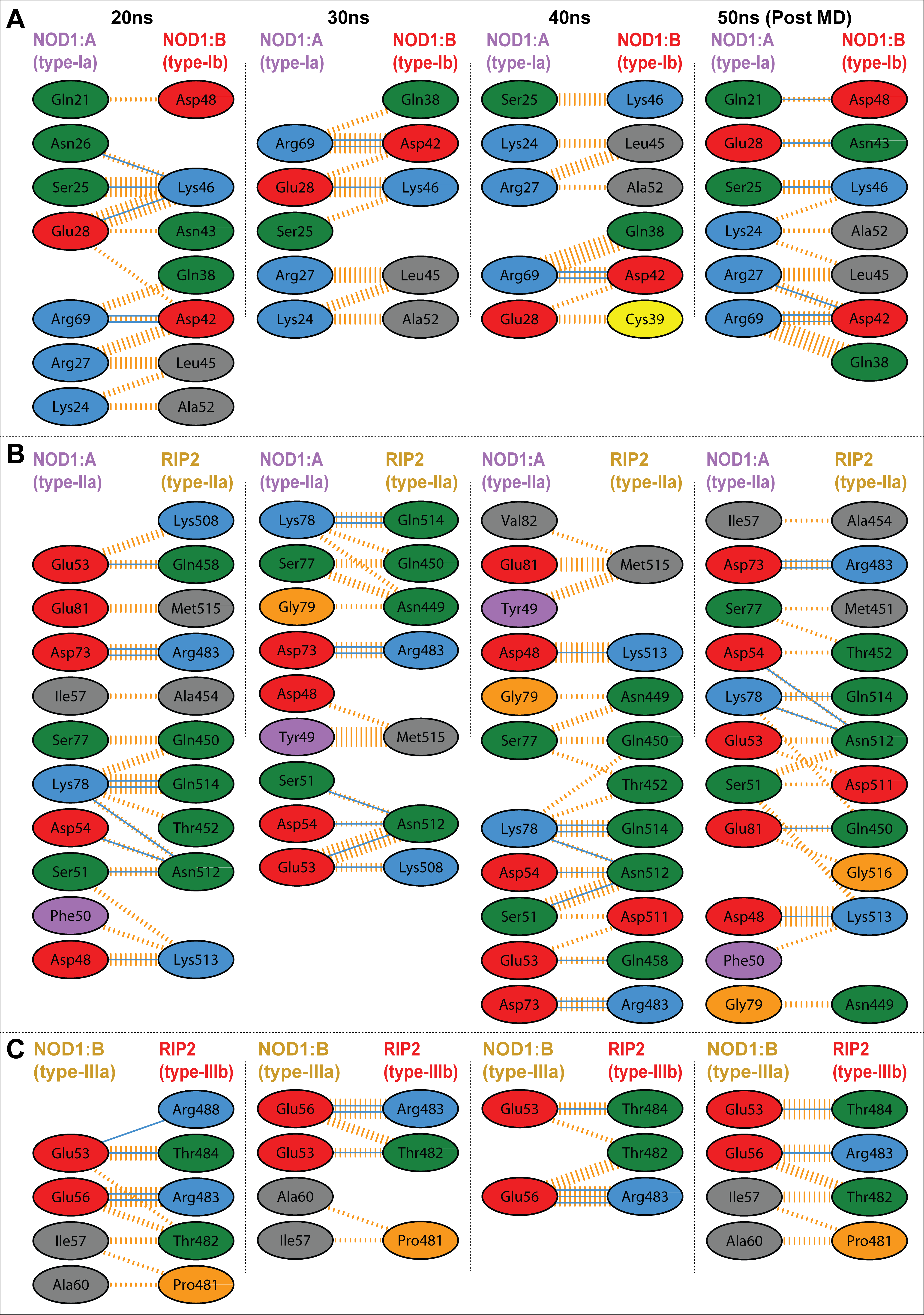

Supplement: S7 Fig — (A) type-I (between two NOD1CARDs); (B) type-II (between NOD1 and RIP2 CARDs); and (C) type-III (between NOD1 and RIP2 CARDs). The molecular interactions were performed using DIMPLOT. The interacting residues are colored according to physicochemical parameters and the blue straight lines and orange dashed lines indicate the H-bonds and hydrophobic interactions, respectively. (TIF) [file pone.0170232.s007.tif]

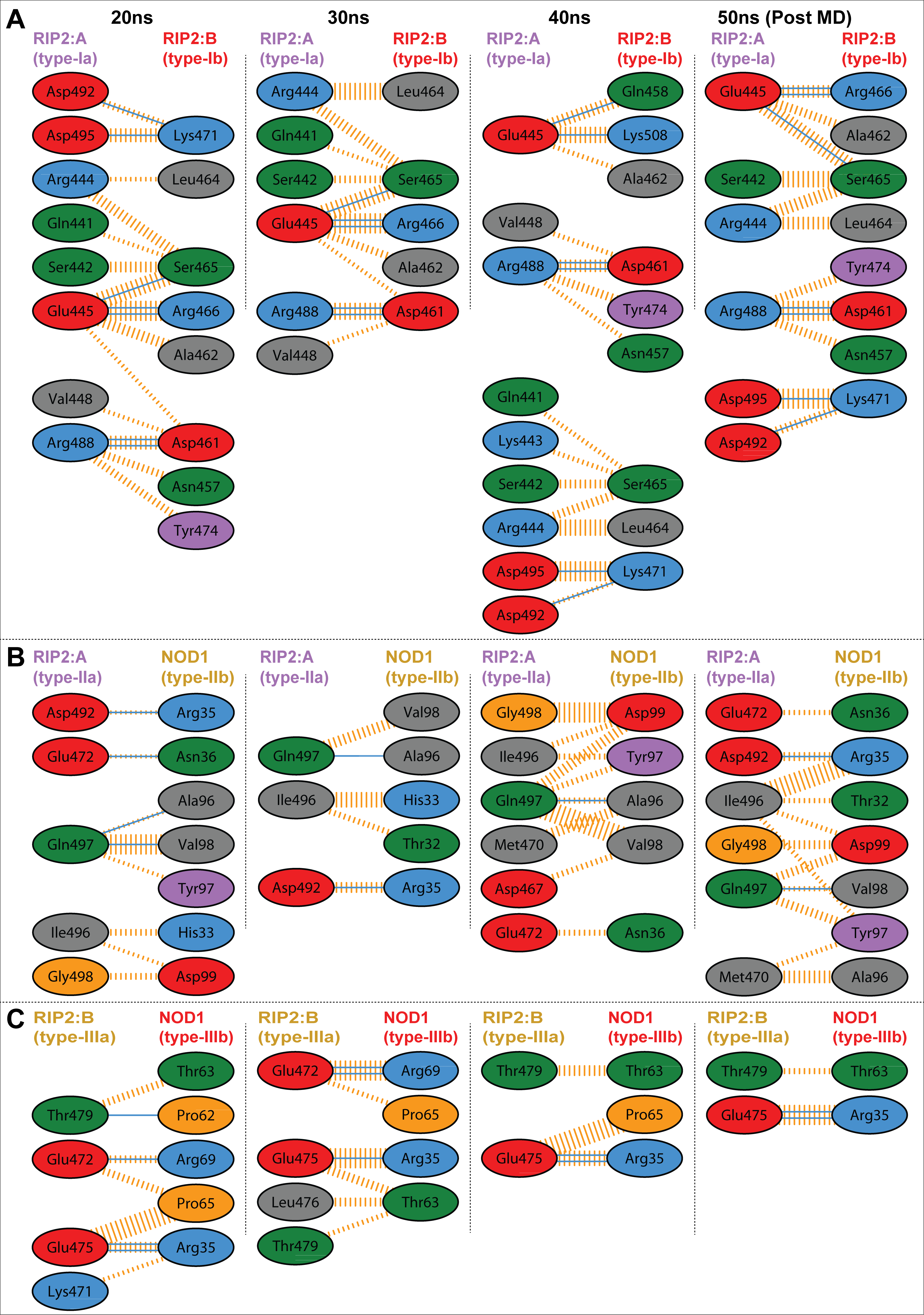

Supplement: S8 Fig — (A) type-I (between two RIP2CARDs); (B) type-II (between RIP2 and NOD1 CARDs); and (C) type-III (between RIP2 and NOD1CARDs). The molecular interactions were performed using DIMPLOT. The interacting residues are colored according to physicochemical parameters and the blue straight lines and orange dashed lines indicate the H-bonds and hydrophobic interactions, respectively. (TIF) [file pone.0170232.s008.tif]

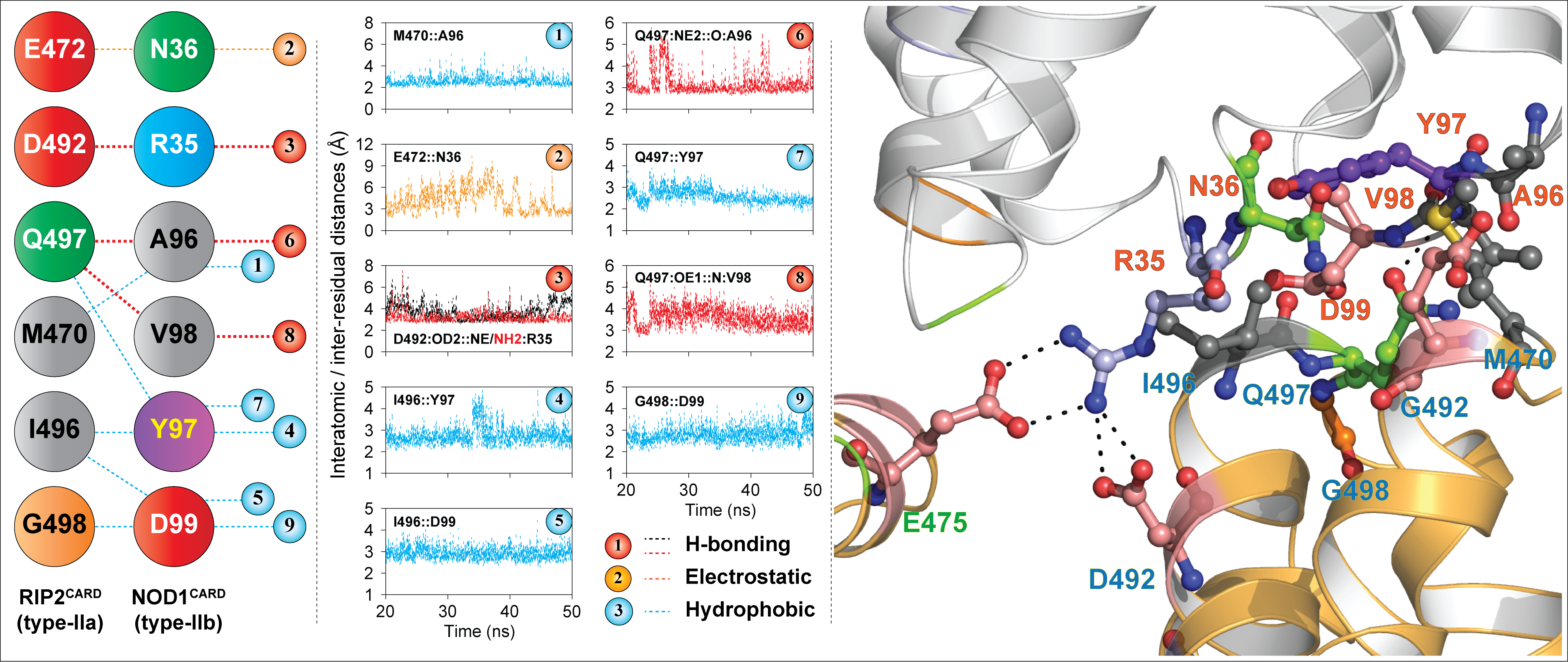

Supplement: S9 Fig — Left panel of the figure shows the conserved interactions (obtained from S9 Fig B) mediated by type-IIa interfaces of RIP2CARD and type-IIb interface of NOD1CARD; the middle panel indicates respective inter-residual/interatomic distances and detailed 3D representation of type-II interaction mode was depicted in the right panel. Critical residues involved in intermolecular interactions were visualized in ball and stick model, and were colored based on physicochemical parameters. The black dotted lines represent the inter/intra-molecular polar contacts, and the labeled blue and orange fonts represent the type-IIa and IIb interface residues. (TIF) [file pone.0170232.s009.tif]
