# Supplementary material for: NOD1CARD Might Be Using Multiple Interfaces for RIP2-Mediated CARD-CARD Interaction: Insights from Molecular Dynamics Simulation
Source: PLoS One. 2017 Jan 23;12(1):e0170232. doi: 10.1371/journal.pone.0170232 (PMC5256935; doi:10.1371/journal.pone.0170232)
Supplement: S1 Table — (DOC) [file pone.0170232.s010.doc]

**Table S1.** Atomic compositions of different simulation systems considered for molecular dynamics simulation.

| **Simulation system** | **No. of water molecule** | **No. of Na^+^** | **No. of Cl^-^** | **Total number of atoms** | **Simulation time (ns)** |
| --- | --- | --- | --- | --- | --- |
| **APO** |  |  |  |  |  |
| NOD1^CARD^ (apo) | 6795 | 25 | 20 | 21948 | 50 |
| RIP2^CARD^ (apo) | 7292 | 23 | 21 | 23408 | 50 |
| **Heterodimers (NOD1-RIP2)** |  |  |  |  |  |
| Complex-I | 15904 | 53 | 46 | 50817 | 50+50+50 |
| Complex-II | 16353 | 54 | 47 | 52166 | 50+50+50 |
| **Homodimeric complexes** |  |  |  |  |  |
| NOD1-NOD1 | 17768 | 61 | 51 | 56452 | 50 |
| RIP2-RIP2 | 14612 | 46 | 42 | 46900 | 50 |
| **Heterotrimeric complexes** |  |  |  |  |  |
| Complex-I (NOD1-RIP2-NOD1) | 22350 | 77 | 65 | 71716 | 60+60+60 |
| Complex-II (NOD1-RIP2-RIP2) | 24419 | 80 | 71 | 77902 | 60+60+60 |
| Complex-III (NOD1-NOD1-RIP2) | 25375 | 89 | 77 | 80815 | 50 |
| Complex-IV (RIP2-RIP2-NOD1) | 22659 | 75 | 66 | 72612 | 50 |
